# Supplementary material for: Adrenocortical Carcinoma in Childhood: A Systematic Review
Source: Cancers (Basel). 2021 Oct 20;13(21):5266. doi: 10.3390/cancers13215266 (PMC8582500; doi:10.3390/cancers13215266)
Supplement: Supplementary file 1 [file cancers-13-05266-s001.zip › cancers-1345218-supplementary.pdf]

SUPPLEMENTARY TABLES

Authors	n ACT	n ACA	Age in years	% Female patients	% Hormone active	% Mixed	% Androgen	% Glucocorticoid	% DOD	Time Interval	Time symptoms-diagnosis (m)	Relapse
Abduch RH 2015	42		2.6	81	100	71	26	2	19	1991-2013	/	/
Almeida MQ 2008	23	17		70						/	/	/
Barbosa AS 2004	8		6.8	50	100	25	63	13	25	/	/	/
Bergada I 1996	20		7.1	75	100	55	45	0	5	1970-1991	10	
Borges KS 2013	60		3.4	77	95					1991-2009	/	/
Brondani VB 2020	36		2.2	64	94	39	53	3	3	/	/	/
Bueno AC 2017	41		15.1	/			17	2	39	1950-2016		10
Bulzico D 2016	27		3.6	70			70	26	37	1997-2015	8	6
Cordeiro AM 2014	39		2.1	67	92	41	49	3			6	/
Damiani D 1995	33		3.7	67	97	/	/	/	/	1975-1993	8	/
Dias AI 2015	27	4	/	56	/	/	/	/	48	1997-2011	/	/
Junqueira T 2012	19		3.3	32					32	1982-2011		
Kremer V 2009	27		3.8	67	93	/	/	/	33	1998-2008	/	/
Latronico AC 2001	18		/	72	100	44	50	0	/	/	/	/
Leal LF 2011	62		1.8	77	100	42	55	3	18	1991-2010	/	7
Leite F 2014	58	13	3.5	78	95	28	64	3	/	1991-2009	/	/
Lira RCP 2016	60		3.5	77	95	30	62	3	/		/	13
Lopes RI 2020	58	43	4.9	71	100	28	64	9	5	1983-2019	/	/
Lorea CF 2012	60		3.5	77	95	30	62	3	2	1991-2009	/	/
Martins-Filho SN 2020	44		2.9	61	98	36	61	5	14	/	/	8
Mastellaro MJ 2018	103		/	67	93	17	73	3	23	1982-2014	4	/
Mendonca BB 1995	18		2.5	72	100	22	72	6	11	1980-1992	/	2
Mermejo LM 2011	62		1.8	77		42	55	3	18			
Michailkiewisc E 1997	20	8	2	95	100	35	65	0	5	1988-1994	6	1
Michailkiewisz E 2004	254	26	3.2	61	90	29	55	6	38	1990-2001	5	/
Monteiro NML 2019	13		2		100	8	0	92	8	2004-2015	10	
Parise IZS 2019	48		2.3	63	83	31	52	0	29	/	/	16
Pereira RM 2013	101		/	/	/	/		/	/	1966-2012	/	23

Pinto EM 2016	86		2.3	73	84	19	55	8	24	/	/	24
Pinto EM 2017	60	10	3.3	70	80	17	38	0	22	2003-2015	/	/
Ribeiro R 1990	40	4	3.9	70	98	55	33	5	28	1966-1987	17	5
Rodriguez-Galindo C 2016	78	/	/	65	/	/	/	/	/	2006-2013		
Sbragia L 2005	33	2	2.3	64	94	21	70	3	12	1980-2004	6	5
Tucci S Jr 2005	34		3	62	97	29	56	12	44	1975-2003	/	13
Venara M 1998	16		7.14	69	100	0	38	63	19	/	/	/
Wajchenberg BL 2000	22		/	/	100	59	41	0	5	1982-1999	/	/
Zancanella P 2006	11		6.3	73	100	27	73	0	73	2003-2004	/	11
**Mean**			**3.88**	**69**	**96**	**33**	**52**	**9**	**22**		**7.9**	
**Median**			**3.3**	**70**	**97**	**30**	**55**	**3**	**19**		**6.8**	
**total**	**1761**											**144**

Suppl. Table S1a: Patient characteristics of the Brazilian Cohort

Descriptive baseline characteristics of 1761 pediatric patients (only Brazilian cohort) with adrenocortical tumor of selected original publications identified by systematic literature review: patient numbers of ACT and ACA, age, % female patients, % hormone active (% mixed, % androgen, % glucocorticoid), %DOD, time interval, time symptoms-diagnosis (months), relapse

Authors	n ACT	n ACA	Age in years	% Female patients	% Hormone active	% Mixed	% Androgen	% Glucocorticoid	% DOD	Time interval	Time symptoms-diagnosis (m)	Relapse	Reported cases of CPS	Country
Benserai FZ 2013	11		/	55	100	/	/	/	/	2002-2013	/	/	/	Algeria
Brenna CTA 2020	23		5.5	78	83	/	26	26	13	1977-2017	/	5	10/20LF	Canada
Buyukpamukcu M 2011	18		5.7	56	89	22	56	11	33	1980-2010	6	/	2SM	Turkey
Cecchetto G 2016	82		8.1	/	31	/	/	/	44	2000-2013	/	40	12CPS	EXPERT (Poland, Germany, France, Italy)
Chatterjee G 2015	13		2.9	69	77	/	/	8	23	2005-2014	/	/	/	India
Chen QL 2011	15		4.3	47	73	40	20	13	/	1991-2010	4	/	/	Taiwan
Chen Y 2017	3		6.8	67	100	/	/	/	/	1990-2015	/	/	2SM	Taiwan
Cho MJ 2012	8		12	38	88	/	25	50	0	1996-2010	6	/	/	Corea
Ciftci AO 2001	20		6.3	55	80	20	30	30	55	1970-1999	8.1	/	/	Turkey
Dall Igna P 2014	58		5.1	67	79	52	/	28	21	1982-2011	/	2	/	Italy
Das S 2016	8		4.3	50	/	/	/	/	38	2005-2016 s.a. Chatterjee	/	11	/	India
Dogman-Bouguerra M 2020	57		5.1	65	88	26	51	5	35		/	13	/	France
Driver CP 1998	14		6	86	100	29	50	29	86	1954-1995	6	/	2/14CPS, 3SM	UK
Federici S 1994	12	4	5	58	100	8	67	25	17	1976-1989	/	Italy	1BW	Italy
Flynt KA 2015	15		12	60	/	/	33	27	1	1995-2014	/	5	/	USA, Ann Arbor
Gönc EN 2018	18		5.7	56	83	28	39	17	22	1999-2017	8	2	1CPS, 1SM	Turkey
Gulack BC 2016	111		4	69	/	/	/	/	/	1998-2011	3	/	/	USA, Duke
Guntiboina VA 2018	12		3.8	50	58	/	/	/	42	2005-2018	/	1	/	India
Gupta N 2018	41		16	78	83	54	17	5	/	1950-2017	3	/	/	USA, Mayo
Hanna AM 2008	23	7	9	65	61	4	57	13	48	1976-2005	/	7	4CPS	USA, Mayo
Hubertus J 2012	59		5.8	68	88	/	80	25	32	1997-2012	/	/	/	Germany
Jehangir S 2019	22	14	7.4	14	/	/	/	/	9	2006-2016	/	/	/	Australia
Kerkhofs TM 2014	12		4.1	67	83	17	50	17	42	1993-2010	/	/	/	Netherlands
Klein JD 2011	29		3.7	59	66	/	55	7	24	1918-2009	/	4	4CPS	USA, Harvard
Knöpfle G 1986	150		4	1	50	19	46	13	/	review	6	14	27/150CPS, 15/150SM	Germany
Letouze E 2012	25		3.5	60	88	28	56	4	28	2001-2015	/	/	21/26CPS	12 Brazil, 13 International
Loncarevic IF 2008	14		45	57	83	14	43	14	43	1998-2008	/	/	1LF	Germany
Magro G 2012	20	13	7.3	60	/	/	35	25	20	2000-2007	/	/	/	Italy
Mahendranaj K 2014	101		7.8	/	/	/	/	/	/	1973-2010	/	/	/	USA, Livingston
Mattone MC 2008	28		4.6	71		57	57	/	/	1987-2017	11	/	/	Argentinia

Mayer SK 1997	11	4	7	73	82	9	55	18	9	1972-1996	18	/	/	Canada
McAteer JP 2013	85		/	67	/	/	/	/	/	1973-2008	/	/	/	USA, Seattle
McDonnell CM 2003	12		2.5	50	58	8	42	8	25	1976-2001	/	/	5/12, 1SM	Australia
Miele E 2020	13		1.4	77	69	46	23	0	0	2010-2020	1	3	6/8LF, 1BW	Italy
Mishra A 2001	10		7.4	60	100	70	10	10	20	1990-1999	14	/	/	India
Mittal R 2012	6		5.5	67	100	/	/	/	83	1989-2009	/	3	/	Kuwait
Narasimhan KL 2003	9		2.5	78	89	44	11	22	11	1989-2000	/	2	/	India
Nazli GE 2014	18		/	/	94	50	/	/	/	/	/	/	/	Turkey
Panamonta O 2001	7	2	/	43	86	29	57	14	29	1986-2000	/	/	1 Down-<br>syndrom	Thailand
Patil KK 2002	21	18	4.9	57	100	86	/	14	10	1972-2000	31	/	1SM	UK
Picard C 2019	95		5	71	78	/	58	23	14	2000-2018	/	13	/	France
Picard C 2020	95		5	71	78	/	58	23	12	2000-2018	/	6	17/55LF,<br>24/95CPS	France
Redlich A 2012	60		5.9	67	88	/	/	/	/	1997-2011	5	37	/	Germany
Ru W 2017	26		4.2	73	92	15	38	38	31	1996-2016	4	8	/	China
Sabaretnam M 2016	16		8	/	88	/	/	/	/	1989-2011	9.2	/	/	India
Sakoda A 2014	29		2.2	52	100	66	17	0	34	1987-2011	6	7	5LF, 3BW	UK
Stewart J 2004	9	1	2.4	56	89	44	44	0	0	1974-2003	6	0	1BW	Canada
Teinturier C 1996	54		4	/	/	/	76	15	/	1973-1993	/	/	15% CPS	France
Waldmann J 2012	2*		16	50	/	/	/	/	/	/	/	/	/	Germany
Wang Z 2019	28	16	2.7	64	75	18	11	4	18	2010-2017	/	7	/	China
Wasserman JD 2015	88		/	68	/	/	/	/	/	/	/	/	32/88LF	USA, St Judes
Wieneke JA 2003	83	9	4	60	82	14	46	20	25	1965-1997	6.8	/	/	USA, Washington
Wolthers OD 1999	30	23	4.9	77	100	/	100	/	13	1976-1996	7	/	/	UK
Wu X 2019	13		3	69	92	23	62	8	/	2009-2018	/	/	8/13LF	China
Yalcin S 2006	24		7.1	/	82	/	/	/	/	1970-2005	9.7	/	/	Turkey
Zekri W 2020	18		4	61	89	6	50	33	44	2007-2016	/	/	/	Egypt
Zerbini C 1992	35		8.1	54	77	17	51	9	46	/	/	/	2LF, 4SM	USA, Washington
**Mean**			**6.5**	**61**	**83**	**31**	**46**	**17**	**28**		**8.46**			
**Median**			**5.05**	**64**	**86**	**26**	**50**	**14**	**25**		**6.00**			
**total**	**1919**	**111**										**190**		
Rodriguez-Galindo 2021**	77		3.2	65	84	/	70	19	/	2006-2013	/	/	41/61 LF	USA, St Judes

ACT= Adrenocortical tumor, ACA= Adrenocortical adenoma, DOD= Died of disease, m= months, CPS= Cancer predisposition syndrome, LF= Li Fraumeni syndrome, SM= Secondary Malignancy , BW= Beckwith-Wiedemann-Syndrome

*2 of 30 patients <18 years

** published after 15th of February 2021 (not included in statistics); patient from Brazil and USA are included

Suppl. Table S1b: Patient characteristics of the non-Brazilian cohort

Descriptive baseline characteristics of 1919 pediatric patients (non-Brazilian cohort) with adrenocortical tumor of selected original publications identified by systematic literature review: patient numbers of ACT and ACA, age, % female patients, % hormone active (% mixed, % androgen, % glucocorticoid), %DOD, time interval, time symptoms-diagnosis (months), relapse, reported cases of CPS, country of population.

Authors	n	I	II	III	IV	unknown/ ACA
Borges KS 2013	60	35	10	8	7	
Brenna CTA 2020	23	9	4	2	7	1
Bulziko D 2016	27	11	5	6	5	
Cordeiro AM 2014	39	22	10	2	5	
Dall Igna P 2014	58	35	17	1	5	
Dias AI 2015	27	11	2	9	5	4 ACA in stage 1
Dogman-Bouguerra M 2020	42	13	12	8	9	
Gupta N 2018	41	2	11	2	26	
Hanna AM 2008	16	2	3	1	10	
Hubertus I 2012	59	5	26	4	24	
Jehangir S 2019	8	1	2	1	4	
Junqueira T 2012	19	3	5	3	8	
Kerkhofs TM 2014	12	6	0	3	3	
Kremer V 2009	27	7	9	5	6	2 ACA in stage 1
Leal LF 2011	62	37	10	8	7	
Leite F 2014	58	34	10	7	7	
Letouze E 2012	25	8	9	6	2	
Lira RCP 2016	60	35	10	8	7	
Loncarevic IF 2008	14	0	5	2	7	
Lorea CF 2012	60	35	10	8	7	
Magro G 2012	20	12	4	1	3	
Martins-Filho SN 2020	44	38	2	2	2	
Mastellaro MJ 2018	103	47	28	20	8	
McAteer JP 2013	85	41	10		28	6 unknown
Mermejo LM 2011	62	37	9	8	8	
Michailkiewisz E 2004	254	112	80	25	37	26 ACA in stage 1
Parise IZS 2019	48	19	14	4	11	
Pereira RM 2013	111	71	40	0	0	
Picard C 2019	95	54	16	18	5	2 unknown
Picard C 2020	95	55	16	17	5	2 unknown
Pinto E 2016	86	28	18	28	12	
Pinto E 2017	60	18	14	13	15	
Redlich A 2012	60	5	26	4	25	
Ru W 2017	26	12	8	5	1	
Sbragia L 2005	33	18	9	3	3	
Stewart JN 2004	8	3	5	0	0	1 ACA in stage 1
Teinturier C 1996	54	42	0	4	7	1 unknown
Tucci S Jr. 2005	34	5	14	5	10	
Wajchenberg BL 2000	23	8	13	0	2	
Wasserman JD 2015	88	27	13	19	14	15 unknown
Wieneke J 2003	83	15	53	11	4	
Zancanella P 2006	11	1	2	3	5	
Zekri W 2020	18	6	4	3	5	
**Mean**	**52**	**23**	**13**	**7**	**9**	
**Median**	**44**	**15**	**10**	**5**	**7**	
**total number of patients**	**2238**	**985**	**568**	**287**	**371**	
**%**		**44**	**25**	**13**	**17**	

Suppl. Table S2: Tumor stage distribution

Descriptive data of tumor stage of 2238 pediatric ACC patients according to the international TNM classification system (stage I, II, III, IV, unknown/ ACA, s. also Table 3 for stage definition) of original publications identified by systematic literature review

Authors	Total of ACC patients	Patients with metastasis	Location of metastasis including lymph node	Only lymph node metastasis N+
Almeida MQ 2008	6	6	/	/
Barbosa AS 2004	8	3	/	/
Bergada I 1996	20	2	lung (2)	/
Brenna CTA 2020	23	6	lung (5), abdominal ((4) liver, Peritoneal)	/
Brondani VB 2020	36	6	/	/
Bueno AC 2017	41	26	lung and liver (4) , lung (3), liver (5), multiple locations (14)	/
Bulzico D 2016	27	5	lung (3), liver (2)	5
Buyukpamukcu 2011	18	7	lung (2), liver (2), lung and liver (2), bones 1	/
Cecchetto G 2016	82	20	kind of metastasis unclear, 11 with LN	5/43
Chatterjee G 2015	13	5	lung (3), liver (2)	/
Chen QL 2011	15	10 (including 4 at the time of relapse)	liver (3), lung (3), retroperitoneal (4)	/
Cho MJ 2012	8	1	liver (1)	/
Ciftci AO 2001	20	10 (including 7 at the time of relapse)	lung (1), liver (1), liver and lung (4), liver and lymph nodes (2), lung and bones (2)	13
Dall Igna P 2014	58	5	/	/
Dias AI 2015	23	5	liver (3), liver and lung (2)	/
Driver CP 1998	14	8	lung (6) , liver (3)	2
Federici S 1994	12	1	/	/
Flynt KA 2015	15	10 (including 3 at the time of relapse)	lung (5) , liver (4), peritoneal (1), all relapses (3) showed multiple locations (lung, lymph nodes,peritoneal)	2
Gönc EN 2018	18	3	liver and lung (2), multiple lesions (1; peritoneal, liver)	/
Gulack BC 2016	111	19	/	/
Gupta N 2018	41	26	liver (19), lung (18)	7
Hanna AM 2008	16	10	liver, bones	/
Hubertus J 2012	59	14	liver	9
Jehangir S 2019	8	4		/
Junqueira T 2012	107	19 (including 11 at the time of relapse)	lung, liver, lymph node	/
Kerkhofs TM 2014	12	3	lung (1), liver (1), lung and liver (1)	/
Knöpfle G 1986	2/150 review	43/150	renal hilus (12), lung (40), liver (20), bones (8), CNS (4), pancreas (1)	5
Kremer V 2009	27	6	/	/
Leal LF 2011	62	7	/	/
Lira RCP 2016	60	20 (including 13 at the time of relapse)	/	/
Loncarevic IF 2008	14	7	/	/
Lorea CF 2012	60	7	/	/
Magro G 2012	7	4 (including 1 at the time of relapse)	lung (2), liver (1), bones and bone marrow (1)	/
Martins-Filho SN 2020	44	2	/	/
Mastellaro MJ 2018	103	8	/	/

Mayer SK 1997	7	1	liver (1)	/
McAteer JP 2013	85	28	/	/
McDonnell CM 2003	12	3	lung (2), liver (1)	/
Mendonca BB 1995	18	2	lung and liver (1), liver and lymph nodes (1)	/
Mermejo LM 2011	62	8	/	
Michailkiewisc E 1997	12	1	lung (1)	/
Michailkiewisz E 2004	228	37	lung (18), liver (11), liver and lung (5), unknown (3)	/
Miele E 2020	13	3	liver, lung	/
Mishra A 2001	10	3	liver (1), LN (2)	2
Mittal R 2012	6	3 (including 1 at the time of relapse)	lung (2), liver (1)	/
Narasimhan KL 2003	9	1	/	1
Panamonta O 2001	7	2	lung (1), liver (1)	/
Parise IZS 2019	48	11	/	/
Patil KK 2002	3	1	/	/
Picard C 2019	95	5	/	3
Picard C 2020	95	5	/	1
Pinto EM 2016	86	12	/	/
Pinto EM 2017	50	15	/	/
Redlich A 2012	60	35(including 23 at the time of relapse)	diagnosis: lung (10), liver(4), LN (3), bones (3), CNS (1); relapse: liver (10), lymph nodes (7), bones (4), CNS (2)	
Ribeiro R 1990	36	5	lung(4), liver and lung (1)	/
Ru W 2017	26	1	/	/
Sakoda A 2014	29	9 (including 7 at the time of relapse)	/	/
Sbragia L 2005	33	6 (including 3 at the time of relapse)	liver (1), lung (4), CNS (1)	/
Stewart J 2004	9	0	/	2
Teinturier C 1996	54	7	/	/
Tucci S Jr 2005	34	12 (including 2 at the time of relapse)	/	/
Venara M 1998	16	2	/	/
Wang Z 2019	12	7	lung (4), liver (2), kidney (1), CNS (1), peritoneal/retroperitoneal (2)	/
Wasserman JD 2015	88	14	/	/
Wolthers OD 1999	7	6	liver (5), lung (5)	/
Yalcin S 2006	24	8	/	/
Zancanella P 2006	11	16 (including 11 at the time of relapse)	diagnosis: lung (3), liver (3), lymph nodes (1); relapse: lung (11), mediastinal (1), kidney (1), liver (3)	/
Zekri W 2020	18	5	/	/
Zerbini C 1992	35	20 (including 17 at the time of relapse)	lung, liver	/

Suppl Table S3: Data and characteristics of metastasis

Data and characteristics of metastasis of pediatric patients with adrenocortical tumor of selected original publications identified by systematic literature review: Total number of ACC patients, number of patients with metastasis, location of metastasis including lymph node, only lymph node metastasis (N+)

Authors	Number of patients	Stage	Pathologic grading: Weiss>3 (W), Wieneke (WI), ENSAT3/4	Tumor size >100g	Tumor size >200cm^3	Age >4 years	Metastasis (M), Lymph nodes (N), Relapse (R)	Tumorextension (T), vascular invasion(V), venous thrombosis (VT)	Surgical outcome: non-R0-resection (NCR), Biopsie (B), tumor rupture (TR)	Hormonal activity	Atyp, mitosis (m), aneuploidie (a), tumor necrosis (n)	High mitotic index (MI), Ki67
Abduch RH 2015 Endc, Reviews	42											
Almeida MQ 2008	23											
Bergada I 1996	20			x							m	MI
Borges KS 2013	60	x	W3	x	x	x						
Brenna CTA 2020	23											MI -
Bueno AC 2017	74											
Bugg MF 1994	54			x	x (>5cm)						a	MI
Bulzico D 2016	27		W3	x	x (>5cm)	x	M+N+R	T	NCR+TR		a- +n	MI-
Chatterjee G 2015	13		WI			x						
Cechetto G 2016	82				x	x	M+N	T+V	NCR	x		
Ciftci AO 2001	20						M		NCR	associated with better prognosis		
Correa CAP 2018	37											
Dall Igna P 2014	58	x		x	x	x			NCR			
Das S 2016	8			x	x			T+V				MI, KI67
Dehner LP 2009	74	x		x							n	
Doghman-Bouguerra 2020	57											
Fedatto PF 2012	34											
Filho SNM 2018	9		W6									
Fragoso MC 2012	24											
Galluzzo Mutti ML 2019	24	x	WI3	x								
Gulack BC 2016	111				x	x	M+N	T+V	NCR			
Guntiboina VA 2018	12			x	x			T+V			m+a+n	MI, Ki67
Gupta N 2018	41	x		x	x	x	M	T+V				
Hanna AM 2008	23		WI3,ENSAT3/4						NCR			KI67 (>20%)
Hubertus J 2012	59			x	x			T+V	B+TR			
Jehangir S 2019	22			x	x			T+V	NCR		m+n	MI
Klein JD 2011	29				x	x					n	MI
Knöpfle G 1986	150						M					
Leal LF 2011	62											
Leite F 2014	45	x		x	x	x						

Lira RCP 2016	60											
Lira RCP 2018	47											
Lorea CF 2012	60	x	W3	x	x	x						
Lucon AM 2002	29		W3									
Mahendraraj K 2014	101	x				x /<5 years (less metastases and lymph nodes)	M+N		NCR			
Martins-Filho SN 2020	44	x	W3WI3			x >3 years				mixed		Ki67<10% =non malign
Mattone MC 2008	28		WI3	x								
McAteer JP 2013	85	x		x	x	x						
Mermejo LM 2011	62											
Michailkiewisz E 2004	20	x		x		x			NCR, TR	virilization better		
Narasimhan KL 2003	9								TR			
Orhan D 2006	30											KI67
Parise IZS 2019	48	x	W3			x>3			NCR			KI67-
Passaia BDS 2018	35					x>5						
Peixoto Lira RC 2016	60											
Pereira RM 2013	101					x		T,V+VT	TR			
Picard C 2019/20	95	x	W3WI3	x	x		M+N	T+V	NCR+B		n	MI, Ki67<15% noM,no relapse
Pinheiro C 2017	50											
Pinto E 2015	37											
Pinto E 2016	86											
Pinto E 2017	60	x		x		x						Ki67 >15%
Redlich A 2012	60	x	W4		x(>300ml)							
Ribeiro R 1990	40			x	x	x						
Ribeiro RC 2004	36		x		x	x				virilization better		
Rodrigues M 2013	48	x				x						
Ru W 2017	26		WI3									
Sakoda A 2014	29	x	W3	x	x (6cm)				NCR + TR			
Sbragia L 2005	33	x			x							
Scridelli CA 2010	57											
Sousa GRV 2014	25											
Sredni ST 2003	24											
Tucci S Jr 2005	34	x			x	x		V				
Venara M 1998	16			(x)	(x)						a	
Wajchenberg BL 2000	47	x			x(>5cm)	x>10y				mixed		

Wang Z 2019	28			x	x			x		Ki67
Wieneke J 2003	83	x	WI3	x	x		V		m+a+n	MI
Yalcin S 2006	34	x					T			
Zekri W 2020	18	x								
Zerbini C 1992	35			x	x	x			a- +n (>25%)	MI

Suppl. Table S4: Factors of poor survival

Factors of poor survival in pediatric ACC of original publications identified by systematic literature review: stage. pathologic grading. tumor size. age >4 years. metastasis (M). lymph nodes (N). relapse. tumor extension (T). vascular invasion(V). venous (tumor-)thrombosis (VTT). surgical outcome. hormonal activity. pathologic criteria. mitotic index

Authors	Number of patients	Surgery	R0	>R0	No Information on extent of surgery	Tumor spillage	Biopsy	Chemotherapy +Surgery	Only Chemo-therapy	Radiotherapy	Mitotane
Bergada I 1996	20	20	/	/	20	/	/	2	/	1	1
Brenna CTA 2020	23	12	/	/	12	/	/	10	0	0	10
Bulzico D 2016	27	27	21	6	/	/	/	8	0	0	13
Buyukpamukcu M 2011	18	13	11	2	/	/	5	4	/	1	6
Checetto G 2016	82	82	31	51	/	/	16	/	/	/	/
Cho MJ 2012	8	8	8	/	/	/	/	/	/	/	/
Ciftci AO 2001	20	17	14	3	/	/	3	2	0	/	14
Dall Igna P 2014	58	58	41	17	/	/	/	6	/	2	11
Dias AI 2015	27	25	23	2	/	10	/	/	/	/	/
Dogman-Bouguerra M 2020	57	55	/	/	55	/	/	27	2	/	/
Driver CP 1998	14	14	3	/	11	/	1	6	/	12	3
Federici S 1994	12	12	1	/	11	/	/	/	/	/	/
Gönc EN 2018	18	15	12	/	3	2	/	3	3	/	5
Gulack BC 2016	111	96	80	16	/	/	/	30	/	/	/
Gupta N 2018	41	37	33	4	/	/	/	21	/	4	/
Hanna AM 2008	23	20	16	4	/	/	/	8	3	2	/
Hubertus J 2012	59	56	31	25	/	11	12	/	/	/	/
Jehangir S 2019	22	/	/	/	/	/	/	6	/	/	2
Junqueira T 2012	19	18	18	/	/	/	/	19	/	/	19
Kerkhofs TM 2014	12	9	8	1	/	/	/	2	/	0	3
Klein JD 2011	29	28	/	/	28	/	/	7	/	3	/
Knöpfle G 1986	150	140	/	/	140	/	/	2	/	17	19
Kremer V 2009	27	17	/	/	17	3	7	18	/	/	8
Leal LF 2011	62	62	/	/	62	/	/	12	/	/	/
Letouze E 2012	25	25	/	/	25	/	/	9	/	/	/
Magro G 2012	20	17	12	5	/	/	1	5	3	/	5
Mastellaro MJ 2018	103	100	72	/	28	/	/	28	3	/	30
Mattone MC 2008	28	?	26	/		/	/	8	/		
Mayer SK 1997	11	10	10	/	/	/	/	2	1	/	/

McAteer JP 2013	85	78	/	/	78	/	/	/	/	8	/
McDonnell CM 2003	12	12	/	/	12	/	/	1	/	/	/
Mendonca BB 1995	18	18	/	/	18	/	/	1	/	/	/
Michailkiewisc E 1997	20	20	20	/	/	1	/	/	/	/	1
Michailkiewisz E 2004	254	254	/	/	254	/	/	65	/	2	80
Miele E 2020	13	13	13	/	/	/	/	2	/	/	7
Mittal R 2012	6	4	4	/	/	/	/	/	1	/	2
Monteiro NML 2019	13	13	12	1	/	1	/	2	/		2
Narasimhan KL 2003	9	9	/	/	9	1	/	/	/	/	/
Panamonta O 2001	7	4	/	/	4	/	2	/	/	/	/
Parise IZS 2019	48	48	46	2	/	/	/	20	/	/	23
Patil KK 2002	21	3	2	1	/	1	/	2	/	1	2
Picard C 2019	95	86	71	14	1	4	9	13	/	1	20
Picard C 2020	95	86	73	13	/	8	5	13	/	1	20
Pinto EM 2017	60	59	36	23	/	10	/	23	1	/	/
Redlich A 2012	60	56	48	8	/	/	/	34	/	7	34
Ribeiro R 1990	40	38	26	6	6	/	6	/	/	/	/
Ru W 2017	26	26	20	6	/	4	/	/	/	/	/
Sakoda A 2014	29	29	22	7	/	7	/	9	/	/	/
Sbragia L 2005	33	33	31	/	2	4	2	/	/	/	/
Stewart J 2004	9	9	9	/	/	2	/	2	0	/	/
Teinturier C 1996	54	54	/	/	54	/	/	11	/	/	20
Tucci S Jr 2005	34	33	32	/	1	/	/	8	/	3	/
Venara M 1998	16	16	16	/	/	/	/	2	/	/	/
Wang Z 2019	28	28	/	/	28	/	/	5	/	/	/
Wieneke JA 2003	83	83	/	/	83	/	/	20	/	8	/
Wu X 2019	13	13	/	/	13	/	/	2	/	/	/
Zancanella P 2006	11	10	7	3	/	/	/	10	1	/	11
Zekri W 2020	18	14	11	3	/	/	/	8	/	/	8
Zerbini C 1992	35	34	/	/	34	/	/	12	/	1	/
**total**	**2221**	**2036**	**976**	**235**	**1009**	**69**	**69**	**510**	**18**	**74**	**360**

Suppl. Table S5: Treatment details

Descriptive data on treatment in 2221 pediatric ACC patients of original publications identified by systematic literature review: radiotherapy, mitotane, chemotherapy and surgery, surgery (R0, >R0), only chemotherapy, tumor spillage, biopsy
